# The Clustering of World Countries Regarding Causes of Death and Health Risk Factors

**Published:** 2018-10

**Authors:** Selen YILMAZ IŞIKHAN, Dilek GÜLEÇ

**Affiliations:** 1. Vocational School of Social Sciences, Hacettepe University, Ankara, Turkey; 2. Dept. of Biostatistics, Faculty of Medicine, Hacettepe University, Ankara, Turkey; 3. Polatlı Vocational School of Health Services, Hacettepe University, Ankara, Turkey

**Keywords:** Death, Risk factors, Cluster analysis, Socioeconomic and sociodemographics indicators

## Abstract

**Background:**

We aimed to determine how many clusters, WHO member countries would be grouped based on the causal rates of disease-specific deaths and preventable risk factors, and evaluated the cluster memberships using some sociodemographic and socioeconomic factors.

**Methods:**

We constructed a dataset relating to 146 WHO countries using reports and some official websites. An explanatory factor analysis was implemented to reveal the underlying patterns of the dataset. The Ward Hierarchical clustering method and gap statistical analyses were used to group countries that have similar causes of death. Clusters were then compared using subgroup analysis based on some socioeconomic and sociodemographic indicators.

**Results:**

We divided 146 countries into six meaningful clusters. In a comparative analysis, the differences between clusters were found to be statistically significant according to disease-specific causes of death, risk factors, socioeconomic, and sociodemographic indicators (*P*<0.001).

**Conclusion:**

Income levels, expenditure rates on health, educational levels, and causes of death in a country are directly proportional to one another. Furthermore, it was surprising that the country clusters regarding causes of death and health risk factors showed regional distributions.

## Introduction

Lifestyle behavior is the cause of about 70%–80% deaths in developed countries (1). Risk factors, such as smoking, alcohol consumption, physical inactivity, obesity, and poor nutrition, are the major causes of mortality that are crucial for researchers and policymakers to improve health and reduce preventable deaths in the developing world (2).

Behavioral risk factors and the associated causes of death are closely linked, and they are affected by the characteristic properties of people and societies. Therefore, the risk factors and related causes of death do not occur randomly and are clustered together. Because of evidence of risk factors’ synergistic effect, there is immense literature on clustering based on multiple risk factors and health behaviors (3). However, little information about which countries might be expected to cluster together based on the causes of death (either disease-specific or behavioral) exists. Clustering of these countries has important implications for developing holistic and preventative health interventions.

This article employed a statistical cluster analysis technique identifying clusters among WHO countries based on causes of death and health risk behaviours and evaluated cluster distributions using socioeconomic and sociodemographic factors.

## Methods

### Data preparation

Cluster analysis was applied to data on the causes of death and risk factors by country, as obtained from the WHO Country Health Profiles (2), World Bank (4), Organisation for Economic Cooperation and Development (OECD) health statistics (5), United Nations Educational, Scientific and Cultural Organization (UNESCO) (6) and International Labour Organisation (ILO) (7) websites. The world’s ten leading causes of death and seven risk factors attributed to global mortality’s leading causes by WHO were used (2). The data preparation process was carefully prepared for 194 WHO member states. Definitions, types, and sources of the variables are as provided below:

### Ten leading causes of death

Ischaemic heart disease, stroke, chronic obstructive pulmonary disease (COPD), lower respiratory infections, trachea bronchus lung cancers, human immunodeficiency virus (HIV)/acquired immunodeficiency syndrome (AIDS), diarrheal diseases, road injury, hypertensive heart disease determine total death percent, and data were obtained from WHO (2), World Bank (4), and UNESCO (6), for 2012–2014.

### Risk factors

The seven risk factors attributed to the leading causes of global mortality included in the analysis are high blood pressure, tobacco use, high blood glucose, physical inactivity, obesity, high cholesterol, and alcohol use. When assigning high blood pressure, the criteria percentages of SBP ≥ 140 and DBP ≥ 90 for people aged 25+ were considered (2). Tobacco was considered as a percent of daily users aged 15+ (2, 7). The percentage of people having high blood glucose levels was determined according to blood glucose ≥ 7 mmol/L or people on medication aged 25+ (2). Insufficient physical activity among adults was included as a percent of inactive people aged 18+ (2, 7). For determining the obesity rate, we considered the percent of people having a BMI > 25 for people aged 20+ (2). Similarly, a raised total cholesterol situation was determined as the percent of people aged 25+ having a value ≥ 200 mmol/L (2). Alcohol use was determined according to the percent of alcohol consumers aged 15+ (2). Risk-factor data was obtained from WHO (2), and ILO (7) reports for the years 2008–2012.

The ten leading causes of death were unavailable for some countries. Missing variables were derived from the *world life expectancy* website (8). The missing death rates were assigned a smaller value (0–0.08) than the death rates that represented the minimum percentage of total deaths in a related country. Twenty-six countries having more than one missing value were excluded from the study, so the data for 146 countries were included in the analysis.

### Socioeconomic and sociodemographic factors

To evaluate clusters, we used some socioeconomic (income, health expenditures, and education) and sociodemographic (region) factors that influence health outcomes. The income variable was grouped into four categories (low, lower-middle, upper-middle, and high) based on the World Bank List (4). The health expenditure variables represented the total expenditures on health as a percent of gross domestic product (GDP), as reported by WHO (2). The education variable considered the mean years of schooling (adults aged 25+) based on the UNDP Human Development Report (9) and the region variable was grouped into six WHO regions: Africa, Americas, Southeast Asia, Europe, Eastern Mediterranean, and Western Pacific, as defined by WHO (2).

### Statistical Analysis

Initially, the dataset’s underlying pattern was determined using a principal component analysis (10). Instead of using all 17 variables, the obtained components were considered in the cluster analysis. The clustering of countries mainly focused on Ward’s algorithm and its dendrogram, and on gap statistics to explore the real clustering structure (10). Ward’s clustering algorithm, termed hierarchical clustering method, clearly separated the countries and assigned the countries to a single cluster based on the related health indicators. Before the multivariate analyses, the outliers for each variable were checked (11,12). First, the squared Euclidean distance as a distance measure, the most commonly used for continuous variables was used (12,13). This is given as follows:

[1] $${\text{D}}_{\text{j}}=\sum_{j=1}^{K}{\left(a_{j}-b_{j}\right)}^{2}$$

In Equation [1], a and b indicate the two cases being compared for the *jth* variable, where *K* represents the total number of variables. At each step in the procedure, the pair of cases or clusters having the smallest squared Euclidean distance are joined with one another (14). This measure also requires that all the indicator variables be on the same scale in the overall distance calculation. Thus, raw data rather than standardized values were used because all variables are measured using a continuous scale (rate).

Second, Ward’s linkage method was used to cluster countries (11). This searching method links the two clusters leading to the smallest increase in the within-cluster sum of squares (12, 15). This method’s advantage compared to other linkage measures (such as a single linkage) is that it is not sensitive to small differences in the data (12).

No strict rule for determining the number of clusters after a hierarchical clustering procedure exist (12). The gap statistic and Ward’s dendro-gram (16) were also utilized for determining the number of clusters. The gap statistic compares the observed and expected values of log W_K_, where W_K_ represents the intra-cluster dissimilarity. It estimates the optimal number of clusters to be the location where the gap between the two curves is largest (17).

Based on the health characteristics, the clusters were introduced; further, a comparison analysis to determine the differences between clusters regarding the socioeconomic indicators (i.e., health expenditures, education, region, and income) using a nonparametric version of the variance analysis (Kruskal-Wallis test) was implemented. Subgroup analysis was performed using a Mann-Whitney U test with a Bonferroni correction. All statistical analyses and plots were implemented with the SPSS 21 (Chicago, IL, USA) program and R programming language version 3.3.1. *P*-values less than 0.05 were considered statistically significant.

## Results

Outlier analysis showed that no outlier rates for the variables exceeded 3%. In the results of the factor analysis, the Kaiser-Meyer-Olkin sampling adequacy measurement was 0.820 and Bartlett’s test of sphericity was statistically significant (*P*<0.01). After a principal component analysis with varimax rotation, five factors were identified, that described the countries’ causes of death and health risk factors. Rotated components and explained total variances are shown in Table 1. These five factors accounted for 73.62% of the total variance. For instance, the countries having high scores of the first principal component also had high rates of alcohol use and cholesterol, trachea bronchus lung cancers, obesity, and tobacco use. Conversely, the countries having low scores were also expected to have low rates for alcohol use, cholesterol, trachea bronchus lung cancers, obesity and tobacco use.

**Table 1:** Factor loadings and explained variance by the five components

***Variables***	***Components***					***Explained Variance (%)***
	***1***	***2***	***3***	***4***	***5***	
Alcohol use	.860					20.00
High cholesterol	.733					
Trachea bronchus lung cancers	.684					
Obesity	.551					
Tobacco use	.421					
Ischaemic heart disease		.852				19.55
Stroke		.848				
High blood glucose		.612				
Lower respiratory inf.		−.588				
Diarrheal disease		−.566				
Phyinactivitiy			.813			12.22
Road injury			.591			
COPD				.823		11.49
Blood pressure				−.724		
HIV-AIDS				−.544		
Diabetes mellitus					.813	10.34
Hypertensive heart disease					.644	
Total						73.62

We determined the clusters based on these five components. Afterward, we examined Ward’s dendrogram and gap statistics to select the shape with the most well-separated clusters; i.e., where the countries within a cluster are similar and differ from the countries in other clusters (10).

A six-cluster solution was obtained through this rule. Table 2 shows the list of countries assigned to the six clusters. Additionally, the dendrogram resulting from this cluster analysis is obtained. If the five-cluster distance is considered, the dendrogram represents six distinct cluster sets.

**Table 2:** List of countries according to the cluster.

***Cluster***	***n (%)***	***Countries***
1	21 (14.4)	Albania, Argentina, Armenia, Azerbaijan, Belarus, Bosnia and Herzegovina, Bulgaria, Croatia, Georgia, Hungary, Kazakhstan, Kyrgyzstan, Latvia, Lithuania, Mongolia, Republic of Moldova, Russian Federation, Slovakia, Turkmenistan, Ukraine, Uzbekistan
2	33 (22.6)	Algeria, Bahrain, Barbados, Brazil, Brunei Darussalam, Colombia, Dominican Republic, Ecuador, Egypt, El Salvador, Guyana, Iran(Islamic Republic of), Iraq, Jordan, Kuwait, Lebanon, Libya, Malaysia, Morocco, Oman, Paraguay, Portugal, Qatar, Republic of Korea, Saudi Arabia, Solomon Islands, Suriname, Syrian Arab Republic, Tunisia, Turkey, United Arab Emirates, Uruguay, Venezuela (Bolivarian Republic)
3	31 (21.2)	Australia, Austria, Belgium, Canada, Chile, Cuba, Cyprus, Czech Rep., Denmark, Finland, France, Germany, Greece, Iceland, Ireland, Israel, Italy, Japan, Luxembourg, Malta, Netherlands, New Zealand, Norway, Poland, Singapore, Slovenia, Spain, Sweden, Switzerland, United Kingdom, United States of America
4	15 (10.3)	Bangladesh, Cabo Verde, Cambodia, China, India, Indonesia, Lao People’s Democratic Republic, Maldives, Myanmar, Nepal, Pakistan, Philippines, Sri Lanka, Thailand, Viet Nam
5	38 (26)	Benin, Botswana, Burkina Faso, Cameroon, CentralAfricanRepublic, Chad, Comoros, Congo, Cote d’Ivoire, DP Congo, Eritrea, Ethiopia, Gabon, Gambia, Ghana, Guatemala, Guinea, Haiti, Kenya, Liberia, Madagascar, Malawi, Mali, Mauritania, Mozambique, Namibia, Niger, Nigeria, Rwanda, Senegal, Sierra Leone, South Africa, Sudan, Swaziland, Togo, United Republic of Tanzania, Zambia, Zimbabwe
6	8 (5.5)	Estonia, Fiji, Jamaica, Lesotho, Mauritius, Mexico, Romania, Trinidad and Tobago

Cluster definitions based on variable characteristics are as follows:
*Cluster 1:* the cluster wherein ischaemic heart disease and stroke are the most frequent causes of death *Cluster 2:* the cluster with the highest rates of inactivity and obesity *Cluster 3:* the cluster wherein high cholesterol, alcohol consumption, and trachea bronchus lung cancers are most frequent *Cluster 4:* the cluster wherein deaths due to COPD are most frequent *Cluster 5:* deaths due to HIV-AIDS and infectious diseases are seen frequently *Cluster 6*: the cluster with the most frequent use of tobacco and most frequent mortality from diabetes and hypertensive heart disease.

Table 3 illustrates the *P*-values of the F-test, where the clusters differ significantly from one another with respect to the causes of death (*P*<0.05). When each variable row was considered, the clusters’ most appropriate names were determined by marking the cells with the highest value. For subgroup analysis’ results, identical symbols were provided to cluster pairs having no statistically significant differences between them. Cluster one, comprising 21 countries, was characterized by ischaemic heart disease (33.50%) and stroke (16.49%) as the leading causes of death; they represented this cluster’s highest percentages.

**Table 3:** Cluster means and F test results

***Variable***	***Cluster1***	***Cluster2***	***Cluster3***	***Cluster4***	***Cluster5***	***Cluster6***	***P***
Alcohol	58.34^*^	26.60^&#^	73.49	23.54^&$^	29.29^#$^	48.7^*^	0.001
Phy. inactivity	23.80^*#+^	38.53	28.59^*^	18.48^&$+^	22.01^*&^^	22.75^#$^^	0.001
Bloodglucose	8.99^*#^	9.63^*$^	7.68^&^^	6.78^^+^	4.75	10.01^#$&+^	0.001
Tobacco	27.42^*#$^	19.17^&^	28.11^*+?^	23.00^#^+^	14.83^&^	29.74^$^?^	0.001
Bloodpressure	28.82^*+^	24.70^#&^	21.22	24.35^#$^	30.26^+^	26.23^*$^	0.001
Obesity	53.29^*#+^	57.46^&^+^	55.93^*$^	21.48	27.58	54.11^#^$^	0.001
Cholesterol	43.96^*&^	43.33^*#^	57.64	33.71^$^	23.61	43.28^&#$^	0.001
Ischaemic heart dis.	33.50	16.12^*#^	14.92^*&+^	11.38^$+^	3.78	15.52^#$^	0.001
Stroke	16.49^+^	10.33^*&#^	8.40^*$^	12.72^&^+^	5.42	9.46^#$^^	0.001
COPD	2.71^*&^	2.32^*#^	3.71^+^	5.37^+^	1.20^$^	1.85^&#$^	0.001
Lower respiratory inf.	2.32^*&^	4.44^#$^	3.67^*#+^	6.98	10.60	3.07^$+^	0.002
Trachea-bronchus lung cancers	3.02^*&^	2.25^#^^	6.38	1.92^*$^+^	0.22	2.06^&#$+^	0.001
HIVAIDS	0.62^*&#^	1.02^*$^	0.12^&^^	1.40^#$^+^	11.75^?^	6.63^+?^	0.001
Diarrheal disease	0.36^*#?^	0.69^$?^	0.40^*^^	2.31^&+^	5.89	0.79^#$^+^	0.001
Diabetesmellitus	1.61^*#^	5.55^*$^	2.51^^+^	3.54^^?^	2.26^&+#^	10.81^$?^	0.001
Roadinjury	1.47^*&^	4.76	1.00^*#^	2.66^$^	2.27^$^^	1.56^&#^^	0.002
Hypertensive heart disease	2.23^*#&^	2.67^*$^+^	2.44^#$^	2.15^&^	1.08	5.82^^+^	0.001

*, &, ^, #, $, + and ? symbols indicate that there is no significant difference between each pair

Alcohol consumption had the second-highest percentage (58.34% of aged 15+) in this group. Cluster two, comprising 33 countries, was characterized by two risk factors: inactivity (38.53% of aged 18+) and obesity (57.46% of aged 25+). Ischaemic heart disease (16.12%) and stroke (9.97%) were the top two causes of death in this cluster; although the rates were smaller than the rates in cluster one. Cluster three, which includes 31 countries, was characterized by alcohol consumption (73.49%) and high cholesterol (57.64% of aged 25+) as the risk factors, and by trachea bronchus lung cancers (6.38%) as the cause of death. Although the mortality rates caused by ischaemic heart disease (14.92%) and stroke (8.40%) were higher, trachea bronchus lung cancers caused the highest mortality rate. Cluster 3 also had the second-highest tobacco usage rate (28.11% of aged 15+). Cluster four, comprising 15 countries, was characterized by COPD, which represented the highest percentage (5.37%) of death from this disease than other clusters. Compared to the other clusters, inactivity (18.48%) and obesity (21.48% of aged 25+) were the lowest risk factors, while lower respiratory infections were the second-highest (6.98%). Cluster five comprising 38 countries was characterized by infectious diseases. HIV-AIDS (11.75%), lower respiratory infections (10.60%), and diarrheal diseases (5.89%) were the three leading causes of death in this group, representing the highest percentages compared to the other clusters. Cluster six, comprising eight countries, was characterized by tobacco use (29.74%) and high blood glucose levels (10.01% of aged 25+) as the risk factors, and diabetes mellitus (10.81%) and hypertensive heart disease (5.82%) as the causes of death.

The percentages of the regional distributions and income levels by cluster, presented in Table 4, show that the country clusters regarding causes of death and health risk factors have regional distributions. Cluster four comprises all Southeast Asian countries, while cluster 5 comprises 89.7% of the African countries. Further, 88.2% of the Eastern Mediterranean countries are assigned to cluster two. Cluster 3 comprises half of the European countries, mostly in Western Europe, whereas cluster one comprises 41.3% of the Middle and Eastern European countries. Western Pacific countries showed more heterogeneous distribution than other regions.

**Table 4:** Cluster percentages across the WHO regions and by income level

***Region***	***Clusters (%)***						***(n)***
	1	2	3	4	5	6	
Africa	0	2.6	0	2.6	89.7	5.1	39
Americas	4.8	52.4	19	0	9.5	14.3	21
Southeast Asia	0	0	0	100	0	0	8
Europe	41.3	4.3	50	0	0	4.3	46
Eastern Mediterranean	0	88.2	0	5.9	5.9	0	17
Western Pacific	6.7	26.7	26.7	33.3	0	6.7	15
Income	***Clusters (%)***						***(n)***
	1	2	3	4	5	6	
Low	0	0	0	4.2	95.8	0	24
Lower-middle	14.7	17.6	0	32.4	32.4	2.9	34
Upper-middle	25.6	41.0	2.6	7.7	10.3	12.8	39
High	12.2	22.4	61.2	0	0	4.1	49

Cluster five includes low-income group countries (95.8%) defined by deaths due to HIV-AIDS and infectious diseases, while cluster three includes high-income group countries (61.2), wherein high cholesterol, alcohol consumption, and trachea bronchus lung cancers are most frequent. Lower and upper-middle income groups show a more heterogeneous distribution than the low and high-income groups.

Table 5 shows health expenditures and education distributions, due to the Kruskal-Wallis test between cluster groups. Health expenditure rates were found to be significantly different among clusters and were, highest in the third cluster (*P*<0.01). Subgroup analysis performed for health expenditures revealed that the 1–3, 2–3, 3–4, 3–5, 3–6, and 1–4 cluster pairs had significant differences between them.

**Table 5:** Health expenditures and education comparisons across clusters

	***Clusters***	***N***	***Mean (sd)***	***Median***	***P***
Health Expenditures	1	21	6.47 (1.87)	6.50	0.001
	2	33	5.88 (1.99)	5.60	
	3	31	9.61 (2.21)	9.70	
	4	15	4.96 (2.90)	4.70	
	5	38	6.02 (2.18)	5.60	
	6	8	6.18 (1.90)	5.75	
	Total	146	6.72 (2.64)	6.35	
Education	1	21	10.41 (1.29)	10.60	0.001
	2	33	7.78 (1.49)	7.70	
	3	31	11.42 (1.02)	11.60	
	4	15	5.90 (2.12)	5.50	
	5	38	4.52 (2.09)	4.25	
	6	8	9.48 (1.87)	9.75	
	Total	146	7.98 (3.12)	8.30	

Similarly, the average years of schooling were significantly different among clusters, being highest in cluster three (*P*<0.01). Subgroup analysis performed for the mean years of schooling revealed that the 1–2, 2–3, 3–5, 3–4, 1–4, and 5–6 cluster pairs had significant differences between them. Table 5 shows that cluster four had the lowest health expenditure rate and cluster five had the lowest education level.

## Discussion

This is the first study examined clusters, causes of death, health risk factors, and socioeconomic and sociodemographic indicators on a globally representative scale. This study reduced ten mortality causes and seven risk-factor variables to five principal components and then performed a cluster analysis utilizing several distance-based algorithms and graphical approaches. Subsequently, we used subgroup analyses to evaluate the clusters. The identified six clusters showed similarities with other studies that classified countries based on health indicators (12,13).

Here, six clusters were identified by classifying 146 countries according to WHO and based on the causes of death and health risk factors. Cluster one included 21 countries, all, except Argentina and Mongolia, in the WHO European region. It is significant that all European countries in this cluster were from Middle and Eastern Europe and mainly had middle-income levels. Ischaemic heart disease and stroke were the highest causes of death compared to the other groups, and tobacco use was 26.73% in this cluster. Marinkovic’s study demonstrated similar findings, indicating that cardiovascular disease represented the highest mortality rate for Eastern and Southeastern European countries (18). Cluster 6 had the highest tobacco use ratio (29.74%). The death rate from ischaemic heart disease was 15.53%, and that of hypertensive heart disease was 5.82%, which was the highest ratio for all clusters.

Physical inactivity increases the risk of ischaemic heart disease, type-2 diabetes, and some cancers (19). Similarly, cluster two was characterized by the highest inactivity and obesity ratios, and the death rate from ischaemic heart disease was 16.04% and that from diabetes mellitus was 5.79%, which were the second-highest ratios compared to the other clusters. 88.2% of Eastern Mediterranean countries, 52.4% of countries in the Americas, and 26.7% of Western Pacific countries were grouped in cluster two, which had the second-lowest health expenditure ratio after cluster four.

Cluster 3, the group with the highest alcohol use rate, mostly included Western European countries. These countries had the highest number of years of schooling, health expenditure ratios, and income levels. Trachea bronchus lung cancer caused the highest mortality rate, and this cluster had the highest cholesterol ratio. Immense evidence demonstrating that there is a negative association between alcohol consumption and death from ischaemic heart disease exists (20, 21). Evidence that alcohol consumption has a positive association with lung cancer and other types of cancer exist (22). Our results also support the existing evidence on the negative association between alcohol consumption and death from ischaemic heart disease. The death rate from ischaemic heart disease was the fourth highest in cluster three, which included countries with high rates of alcohol consumption. Smoking is responsible for a third of all cancer deaths in many Western countries (23). Thus, this group’s tobacco use ratio was 28.11, which is only slightly below cluster six, which had the highest tobacco use ratio (29.74%).

Cluster four demonstrated the lowest health expenditure ratio, characterized by the highest death rate from COPD. Most of the lower-middle income group and all of the Southeast Asian countries were included in this cluster. The inactivity and obesity rates were the lowest and the lower respiratory infection rate was the second highest among the clusters.

Cluster five, characterized by infectious diseases, included 95.8% of low-income countries and 89.7% of African countries. This group had the lowest number of years of schooling. Three leading causes of death in this group were HIV-AIDS, lower respiratory infections, and diarrheal diseases. Evidence regarding the association between socioeconomic conditions and mortality exists (24). This cluster may represent the interaction between poor socioeconomic status and causes of death.

Cluster six, comprising eight countries, was the most heterogeneous group with regards regional positions and income levels. The group was characterized by the highest tobacco use rate and highest death rate from hypertensive heart disease. Many studies have shown large associations between the individual and social determinants of health and mortality (25). Thus, income levels, health expenditure rates, time spent in school, and causes of death in a country are directly proportional. Our study demonstrated that the leading causes of death are infectious diseases in lower-income countries and ischaemic heart disease, stroke, and trachea bronchus lung cancers in higher income countries.

The six clusters based on the causes of death showed regional distributions, similar to Marinkovic’s study, indicating the significance of regional factors on the causes of death (18). Similarly, health mostly depends on geography and development (12). These findings led to our deliberations about the interactions between ethnicity, culture, environmental determinants, and mortality. Geographic indicators may have powerful effects on the causes of death.

The limitation of the study is exclusion of 26 countries with missing data from the analysis. On the other hand, considering more than one statistical approach to determine the number of clusters and examining the changes in different indicators by clusters using subgroup analyses is the strength of the study.

## Conclusion

The six clusters, obtained by cluster analysis of WHO countries based on causes of death and health risk factors, have significant differences according to various health characteristics. Regional distribution of countries into clusters is very remarkable. This finding made us think the importance of climate and ethnicity rather than socioeconomic factors in the cluster separation. We need more comparative studies on the causes of death, using geographical variables such as ethnicity, culture, climate, and environmental determinants.

## Ethical considerations

Ethical issues (Including plagiarism, informed consent, misconduct, data fabrication and/or falsification, double publication and/or submission, redundancy, etc.) have been completely observed by the authors.
